# Nomogram for predicting overall survival in patients with triple-negative apocrine breast cancer: Surveillance, epidemiology, and end results-based analysis

**DOI:** 10.1016/j.breast.2022.08.011

**Published:** 2022-09-02

**Authors:** Yinggang Xu, Weiwei Zhang, Jinzhi He, Ye Wang, Rui Chen, Wenjie Shi, Xinyu Wan, Xiaoqing Shi, Xiaofeng Huang, Jue Wang, Xiaoming Zha

**Affiliations:** a Department of Breast Disease, The First Affiliated Hospital of Nanjing Medical University, No.300 Guangzhou Road, Nanjing, 210000, China; b Collaborative Innovation Center for Cancer Personalized Medicine, Nanjing Medical University, Nanjing, 210000, China

**Keywords:** Breast cancer, Nomogram, SEER, Survival

## Abstract

**Purpose:**

Triple-negative apocrine carcinoma (TNAC) is a sort of triple-negative breast cancer (TNBC) that is rare and prognosis of these patients is unclear. The present study constructed an effective nomogram to assist in predicting TNAC patients overall survival (OS).

**Methods:**

A total of 373 TNAC patients from the surveillance, epidemiology, and end results (SEER) got extracted from 2010 to 2016 and were divided into training (n = 261) and external validation (n = 112) groups (split ratio, 7:3) randomly. A Cox regression model was utilized to creating a nomogram according to the risk factors affecting prognosis. The predictive capability of the nomogram was estimated with receiver operating characteristic (ROC) curve, calibration curve, and decision curve analysis (DCA).

**Results:**

Multivariate Cox regression analysis revealed age, surgery, chemotherapy, stage, and first malignant primary as independent predictors of OS. A prediction model was constructed and virtualized using the nomogram. The time-dependent area under the curve (AUC) showed satisfactory discrimination of the nomogram. Good consistency was shown on the calibration curves in OS between actual observations and the nomogram prediction. What's more, DCA showed that the nomogram had incredible clinical utility. Through separating the patients into groups of low and high risk group that connects with the risk system that shows a huge difference between the low-risk and high risk OS (*P* < 0.001).

**Conclusion:**

To predict the OS in TNAC patients, the nomogram utilizing the risk stratification system that is corresponding. These tools may help to evaluate patient prognosis and guide treatment decisions.

## Highlights

• We developed a nomogram to predict 3-, 5-year OS for stage Triple-negative apocrine cancer.
• Predictive accuracy of the nomogram was superior to that of the traditional classification.
• This tool will be useful for predicting the OS of TNAC patients and selecting individualized treatment.

## 1 Introduction

Apocrine carcinoma (AC) is an intriguing kind of breast cancer described by the multiplication of enormous abnormal cells with rigorously characterized borders, plentiful eosinophilic cytoplasm, huge cores, and noticeable nucleoli that distinguish it from non-AC [1]. AC was first described by Krompecher in 1961 and was recognized as a distinct type of breast cancer by the World Health Organization in 2019 [2]. It is similar to apocrine sweat glands and is characterized by >90% of cells with apocrine morphology [2,3].

AC represents 0.3%–4% of all breast cancers [1,4]. It typically presents as estrogen receptor (ER)- negative, progesterone receptor (PR)- negative, and androgen receptor (AR)- positive. Overexpression of human epidermal advancement factor receptor 2 (HER2) addresses around 30% of ACs; accordingly, most ACs are triple-negative [3,[5], [6], [7]].

Triple-negative apocrine carcinoma (TNAC) is an uncommon kind of triple-negative breast cancer (TNBC) that records for around 1%; hence, the prognosis of these patients is accounted for in a set number of case reports or studies that enrolled not many TNAC patients. Subsequently, the forecast of patients with TNAC stays indistinct. A few studies have recommended that patients with TNAC present with lower grade and stage and are common in elderly women [8,9]. TNAC was also reported in two studies to have favorable overall survival (OS) compared with other TNBC tumors [10,11]. However, the small sample size of these studies provided limited prognostic information. In this manner, it is critical to explain the clinicopathological qualities and visualization of TNAC in a huge populace.

The Surveillance, Epidemiology, and End Results (SEER) program is an expansive populace based data set for malignant growth related the study of disease transmission and wellbeing related help research. It incorporates information from 18 topographically factor populace based malignant growth libraries, which cover practically 30% of the number of inhabitants in the USA [12]. Nomograms are generally utilized as a straightforward and dependable prescient device for the prognostic assessment of numerous tumors. They coordinate different significant factors and convert the measurable expectation model into a solitary mathematical gauge of the likelihood of an occasion, like the likelihood of endurance or passing from an illness, as an outline [13]. As a result, nomograms have turned into a solid device to direct navigation and foresee clinical results for some diseases.

The present study aimed to establish and validate a new prediction model to predict future TNAC patients using the cohorts in the SEER database.

## 2 Materials and methods

### 2.1 Patients and selection criteria

The SEER data set (https://seer.cancer.gov/) incorporates 18 populace based tumors. All the data for patients with TNAC was removed from the SEER data set utilizing the SEER*Stat program (v 8.4.0). The conditions for extraction were according to the following: ''the location of the disease: breast,'' '' diagnosis year: 2010–2016,'' “age at diagnosis: ≥18,” and “breast subtype: HR-/HER2-." The accompanying factors were removed: patient ID, age at analysis, ethic recode, sex, stage, ICD-*O*-3 histology/conduct, 6th edition American joint Committee on Cancer (AJCC) classification, chemotherapy recode, radiation recode, code to site recode, survival months, surgery performed, and first malignant primary site. The exclusion criteria were: unclear cause of death; unknown AJCC tumor–node–metastasis (TNM) stage; and unclear surgery performed. In total, there were 377 TNAC patients and 36,924 non-TNAC TNBC patients. A final total of 373 TNAC patients were signed up for the current review and haphazardly partitioned into one or the other preparation (n = 261) or external validation (n = 112) groups (split ratio, 7:3). The training group was utilized to lay out the prescient model and develop the nomogram and risk stratification system. Information from the external validation group were utilized to verify the model.

### 2.2 Development of the nomogram

OS was surveyed utilizing Kaplan-Meier analysis and contrasted with log-rank test analysis. The duration time between surgery and death is defined as OS. The connection between clinicopathological elements and OS was surveyed utilizing Cox proportional hazards regression model, and hazard ratio (HRs) and corresponding 95% confidence intervals (CIs) were determined. Univariate Cox proportional hazards regression model was utilized to survey the prescient capacity of every boundary. Factors with *P*-values <0.05 in the univariate analysis were additionally analyzed in the multivariate Cox regression model. In light of the consequences of multivariate analysis, the chose independent risk indicators were integrated into the nomogram to anticipate the likelihood of 3 and 5-year OS rates using R 4.1.3 statistical software (http://www.rproject.org).

The AUC mirrors the expectation exactness and separation capacity of the new created nomogram. DCA was a method performed to evaluate the clinical benefits of the new nomogram. Risk stratification system was created in accordance with the total score of each and every patient in the training group. All patients were apportioned into two prognostic social affairs: low or high risk group. Kaplan-Meier curve analysis and log-rank test were utilized to address and dissect the OS of the patients.

### 2.3 Statistical analysis

Information were examined utilizing R programming (R 4.1.3, http://www.rproject.org) and IBM SPSS Statistics (IBM Corp, v26.0, Armonk, NY, USA). All proofs were two-way. *P*-values <0.05 were thought of as huge for any remaining tests.

## 3 Results

### 3.1 Patient attributes

A total of 373 eligible patients were identified from the SEER database between 2010 and 2016. TNAC was confirmed in all cases at the time of diagnosis. Table 1 summarizes the clinicopathological features and treatment experience of all patients. The median age of all patients was 67 years. All eligible cases were randomly assigned to either the training (261, 70%) or external validation (112, 30%) groups. The baseline characteristics were displayed in Table 1.

**Table 1:** Demographics and clinicopathologic characteristics of the training and external validation groups.

Characteristics	All patients (n = 373), n (%)	Training group (n = 261), n (%)	External validation group (n = 112), n (%)
Age (years)			
Median	67 (24–97)	68 (24–97)	65 (35–95)
≤50	35 (9.38)	25 (9.58)	10 (8.93)
>50	338 (90.62)	236 (90.42)	102 (91.07)
Ethnicity			
White	283 (75.87)	201 (77.01)	82 (73.21)
Black	49 (13.14)	37 (14.18)	12 (10.71)
Other	39 (10.46)	23 (8.81)	16 (14.29)
Unknown	2 (0.54)	0	2 (1.79)
Tumor			
T1	233 (62.47)	169 (64.75)	64 (57.14)
T2	105 (28.15)	70 (26.82)	35 (31.25)
T3	23 (6.17)	14 (5.36)	9 (8.04)
T4	12 (3.22)	8 (3.07)	4 (3.57)
Node			
N0	255 (68.36)	181 (69.35)	74 (66.07)
N1	82 (21.98)	57 (21.84)	25 (22.32)
N2	19 (5.09)	15 (5.75)	4 (3.57)
N3	17 (4.56)	8 (3.07)	9 (8.04)
Metastasis			
M0	363 (97.32)	257 (98.47)	106 (94.64)
M1	10 (2.68)	4 (1.53)	6 (5.36)
Stage			
I	185 (49.60)	132 (50.57)	53 (47.32)
II	138 (37.00)	97 (37.16)	41 (36.61)
III	40 (10.72)	28 (10.73)	12 (10.71)
IV	10 (2.68)	4 (1.53)	6 (5.36)
Primary site			
Central portion	20 (5.36)	13 (4.98)	7 (6.25)
Upper–inner	38 (10.19)	27 (10.34)	11 (9.82)
Lower–inner	17 (4.56)	14 (5.36)	3 (2.68)
Upper–outer	141 (37.80)	100 (38.31)	41 (36.61)
Lower–outer	29 (7.77)	19 (7.28)	10 (8.93)
Nipple	2 (0.54)	1 (0.38)	1 (0.89)
Overlapping	97 (26.01)	67 (25.67)	30 (26.79)
Unknown	29 (7.77)	20 (7.66)	9 (8.04)
Surgery			
BCS	199 (53.35)	138 (52.87)	61 (54.46)
Mastectomy	166 (44.50)	118 (45.21)	48 (42.86)
No surgery	8 (2.14)	5 (1.92)	3 (2.68)
Chemotherapy			
Yes	223 (59.79)	155 (59.39)	68 (60.71)
No/unknown	150 (40.21)	106 (40.61)	44 (39.29)
Radiotherapy			
Yes	186 (49.87)	123 (47.13)	63 (56.25)
No/unknown	187 (50.13)	138 (52.87)	49 (43.75)
First malignant primary			
Yes	317 (84.99)	220 (84.29)	97 (86.61)
No	58 (15.55)	41 (15.71)	15 (13.39)

BCS, Breast-conserving surgery.

In the training group, 236 (90.42%) patients were aged >50 years and 229 (87.73%) patients were stage I–II. Among these patients, 138 (52.87%) patients underwent breast-conserving surgery and 118 (45.21%) underwent mastectomy. Furthermore, 155 (59.39%) patients received chemotherapy and 220 (84.29) patients were categorized as first malignant primary tumor. In the external validation group, 102 (91.07%) patients aged >50 years and 94 (83.93%) patients were stage I–II. Among these patients, 61 (54.46%) patients underwent breast-conserving surgery and 48 (42.86%) underwent mastectomy. In addition, 68 (60.71%) patients received chemotherapy and 97 (86.61) patients were categorized as first malignant primary tumor.

### 3.2 Univariate and multivariate analysis and distinguishing proof of prescient variables

Cox proportional hazards model analysis was conducted to examine the power of each indicator in anticipating OS for the training group. Univariate analysis identified indicators including age, ethnicity, TNM stage, stage, tumor primary site, surgery performed, chemotherapy, radiation therapy, and first malignant primary. Factors with a *P*-value <0.05 in the univariate analysis were additionally inspected in the multivariate analysis utilizing a backward model strategy (*P* > 0.10). At long last, factors including stage, surgery, chemotherapy, and first malignant primary were recognized as independent indicators of OS and remembered for the prescient model. Age was likewise thought to be as it is a independent indicator for breast cancer (Table 2).

**Table 2:** Univariate and multivariate analysis of overall survival (OS) in the training group.

Variable	Univariate analysis		Multivariate analysis	
	HR (95% CI)	*P*-value	HR (95% CI)	*P*-value
Age (years)	1.841 (0.442–7.672)	0.402	2.946 (0.577–15.046)	0.194
≤50				
>50				
Ethnicity		0.005		0.183
White			1.0 (reference)	
Black	0.295 (0.071–1.232)	0.094	0.231 (0.049–1.097)	0.065
Other	<0.001	0.973		0.977
Unknown				
Tumor		0.076		
T1	1.0 (reference)			
T2	1.181 (0.5534–2.611)	0.681		
T3	3.446 (1.286–9.237)	0.014		
T4	2.382 (0.554–10.238)	0.243		
Node		0.002		
N0	1.0 (reference)			
N1	1.755 (0.788–3.909)	0.168		
N2	1.145 (0.265–4.941)	0.856		
N3	8.640 (3.423–21.81)	<0.001		
Metastasis	30.111 (9.985–90.809)	<0.001		
M0				
M1				
Stage		<0.001		<0.001
I			1.0 (reference)	
II	1.212 (0.543–2.706)	0.639	1.582 (0.655–3.819)	0.308
III	2.412 (0.962–6.048)	0.061	2.949 (1.043–8.336)	0.041
IV	37.262 (11.456–121.196)	0.000	139.74 (29.329–655.639)	<0.001
Primary site		0.079		
Central portion	1.0 (reference)			
Upper–inner	0.324 (0.054–1.945)	0.218		
Lower–inner	1.162 (0.234–5.774)	0.855		
Upper–outer	0.386 (0.104–1.427)	0.153		
Lower–outer	0.513 (0.086–3.070)	0.465		
Nipple	<0.001	0.978		
Overlapping	0.511 (0.135–1.929)	0.322		
Unknown	1.701 (0.451–6.415)	0.433		
Surgery		0.001		0.001
BCS	1.0 (reference)		1.0 (reference)	
Mastectomy	2.601 (1.231–5.494)	0.012	1.363 (0.575–3.23)	0.482
No surgery	16.106 (4.386–59.139)	<0.001	14.19 (3.495–57.613)	<0.001
Chemotherapy	2.191 (1.113–4.311)	0.023	3.136 (1.396–7.049)	0.006
Yes				
No/unknown				
Radiotherapy	0.712 (0.498–1.017)	0.062		
Yes				
No/unknown				
First malignant primary	0.462 (0.329–0.649)	<0.001	4.129 (1.911–8.918)	<0.001
Yes				
No				

BCS, Breast-conserving surgery.

### 3.3 Building and approving the original nomogram

**Fig. 1:**
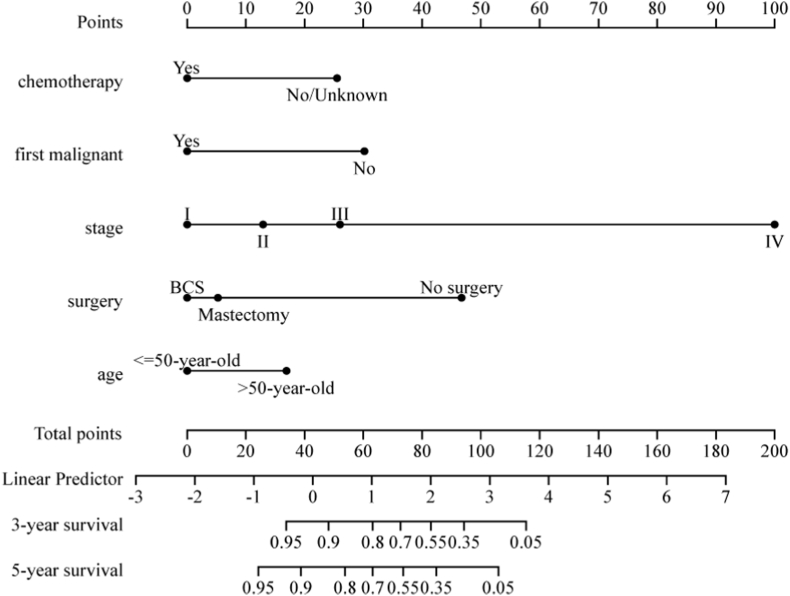
Prognostic nomograms of 3 and 5-year overall survival.

The prescient model was basically introduced as a nomogram (Fig. 1) and approved utilizing the external validation group.

**Fig. 2:**
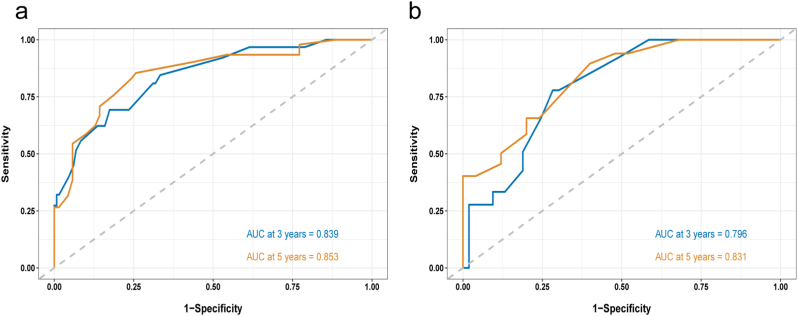
**ROC curve analysis.** ROC curves and AUCs at 3 and 5 years in the training (a) and external validation (b) groups were utilized to gauge the prognostic precision of the nomogram.

The novel nomogram revealed that the AUC at 3 years was 0.839, whereas the AUC at 5 years was 0.853, reflecting the model's good ability to discriminate (Fig. 2).

**Fig. 3:**
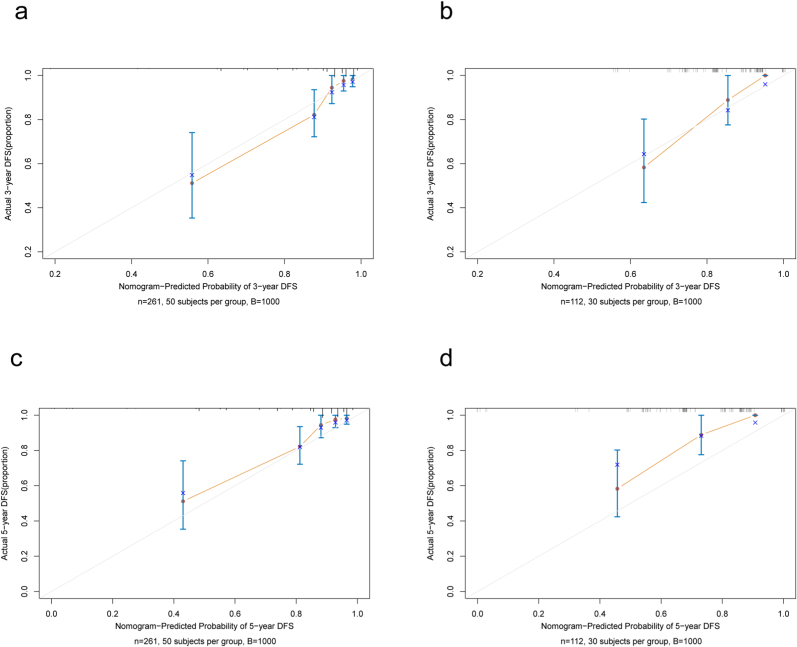
**Calibration curves foreseeing the 3 and 5-year OS of patients.** Calibration foreseeing the 3 and 5-year OS of patients in the training (a, c) and external validation (b, d) groups. The x-axis shows the predicted survival likelihood and the y-hub demonstrates the real survival likelihood. The 45-degree line (dim line) shows that the expectation concurs with real result.

Calibration curves additionally showed great consistency in the likelihood of 3 and 5-year OS between the genuine observation and nomogram expectation (Fig. 3).

**Fig. 4:**
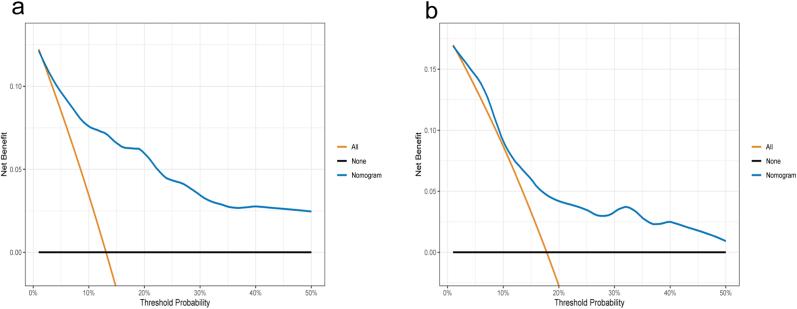
**Decision curve analysis (DCA) of the nomogram predicting OS.** Decision curves of the nomogram foreseeing OS in the training (a) and external validation (b) group. The x-axis addresses the limit probabilities and the y-axis estimates the net advantage determined by adding the true positives and subtracting the false positives. The level line along the x-axis expects that OS happened in no patients, though the solid dark line expects that all patients will die at a particular limit likelihood. Dashed line addresses the net advantage of utilizing the nomogram.

Moreover, DCA displayed solid positive net advantages in the prescient model among practically all of the threshold probabilities at various time focuses, demonstrating a great likely clinical impact of the prescient model (Fig. 4).

### 3.4 Risk stratification framework

**Fig. 5:**
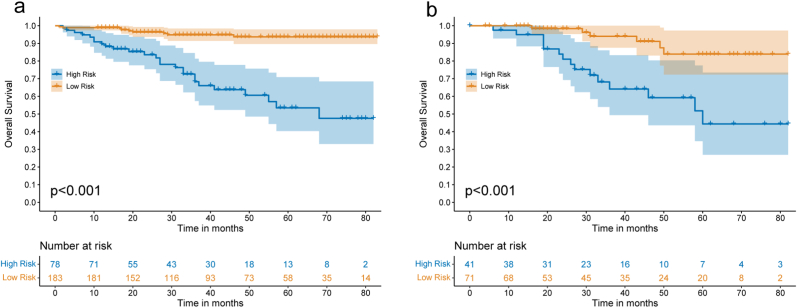
**Kaplan–Meier curves of OS for risk stratification.** Kaplan–Meier curves of OS for risk stratification in the training (a) and external validation (b) groups.

Apart from the nomogram, a risk stratification system for OS was created in reference to the scores of patient in the development partner delivered by the nomogram to partition all patients separately into two groups utilizing an optimum cutoff value. Founded on the novel stratification system, patients in the training group were characterized into low-risk (183/261, 70.1%) or high-risk (78/261, 29.9%) groups (*P* < 0.001, Fig. 5). Kaplan-Meier curves showed that the OS in the various groups was precisely separated by the risk stratification system (Fig. 5a). It also reflected the predictive power of the model in the external validation group (*P* < 0.001, Fig. 5b).

## 4 Discussion

AC is a rare type of breast cancer that is usually ER-negative, PR-negative, and AR-positive, and 30% show amplification of HER2 [[14], [15], [16]]. As a result, 70% of ACs are triple-negative breast cancers. Although TNBC is generally considered an aggressive breast cancer, studies have shown that TNAC has a significantly better prognosis than other TNBCs [10,11,17,18]. The present study used the SEER database to identify 377 patients diagnosed with TNAC and 36,924 patients diagnosed with TNBC between 2010 and 2016. Kaplan–Meier analysis was used to evaluate OS, which was compared utilizing log-rank test (Fig. S1).

Our discoveries showed that TNAC patients had a superior OS contrasted with TNBC patients (*P* < 0.001). However, treatment for TNAC still follows the treatment for invasive ductal carcinoma. Considering the better prognosis of TNAC compared with TNBC, individualized treatment and de-escalation therapy for TNAC patients are worth considering. It is necessary to predict the prognosis of patients with TNAC and guide treatment; therefore, we constructed a nomogram to predict the long-term survival of TNAC patients based on the large-sample database of the SEER program. The nomogram combined conventional available information, such as age, stage, surgery, chemotherapy, and first malignant primary, to predict OS in 373 TNAC patients.

The 373 patients included in the study were randomly allocated to training and external validation groups (ratio of 7:3) on the basis of the SEER database. Our outcomes showed that calibration of the nomogram accomplished astounding consistency between the training and external validation groups (Fig. 3). The AUC at 3 years (0.839) and 5 years (0.853) in the training groups were also high enough to verify the discrimination of the nomogram model (Fig. 2a). The AUC value of the external validation was also sufficient (Fig. 2b). DCA demonstrated the clinical benefits and practicability of our nomogram for predicting OS compared with traditional assessment systems (Fig. 4).

Univariate and multivariate analyses revealed four independent risk indicators, including stage, surgery, chemotherapy, and first malignant primary tumor. We included age as a risk indicator in the nomogram model since age is an independent predictor of breast cancer [19], even though age was not significant in the univariate or multivariate analyses. These independent prognostic indicators identified in our study are in agreement with previous studies [10,11,17,20]. The present study found that TNAC patients were older, had lower tumor levels, and had a lower incidence of T-stage tumors than TNBC patients [8,9,18]. The greater part of these discoveries are reliable with past studies. These indicators are generally recognized as reliable prognostic indicators [[21], [22], [23], [24]]. According to our nomogram, TNAC patients aged <50 years and with lower level of stage have a better prognosis; therefore, de-escalation therapy may be appropriate for these patients. TNAC is usually grouped with other TNBCs due to a lack of accurate prognostic data and usually relies on efficient, multicomponent chemotherapy. Furthermore, TNAC patients are usually elderly with low tumor levels and are unable to receive chemotherapy. Only 223 patients received chemotherapy in the present study, accounting for 59.79% of all enrolled patients. However, the findings of the present study indicate that chemotherapy can improve the prognosis of patients. Chemotherapy has been reported to benefit TNAC patients [25], although the current standard treatment is either total mastectomy or breast-conserving surgery plus radiotherapy [26]. The two methods have shown their equivalence in recurrence-free rate and OS. In the present study, surgery improved the OS of TNAC patients compared with no surgery, although breast-conserving surgery showed no significant advantage over mastectomy.

A nomogram was laid out in view of five independent risk indicators and patients were isolated into high-risk and low-risk groups in reliance on ROC curve analysis. Cheeringly, the low-risk group showed fundamentally preferred OS over the high-risk group (*P* < 0.001, Fig. 5). Nomograms may be better at identifying high-risk groups compared with the traditional stage system.

As far as anyone is concerned, this is the main review to report of the utilization of a nomogram to foresee OS in TNAC patients utilizing the SEER data set. We analyzed a large sample of 18 medical centers registered in the SEER database. The time-dependent AUC and calibration curves were calculated using cross-validation methods. Analysis of the validation group replicated the positive results, implying that the nomogram can be used to assess patient prognosis. In general, chemotherapy and surgery can prolong the OS of patients with TNAC, even if the prognosis is better than that of TNBC, and age <50 years, low stage, and first malignant tumor indicate better prognosis. Therefore, we can classify TNAC patients according to these five risk indicators into high-risk and low-risk groups and evaluate their prognosis. In addition, de-escalation therapy can also be used for young, low-stage patients.

The current review has a few restrictions. In the first place, this was a retrospective analysis of the SEER data set; therefore, the study's validity may be subject to selection bias. Second, the stage was based on the AJCC 6 staging system, which may reduce efficiency. Third, several patients in the study lacked clear details about chemotherapy and/or radiation therapy due to coding restrictions in the SEER database. Since we split prevalence into treated and untreated patients, the categorical variables' statistical power may have been reduced. Finally, because TNAC is a rare form of breast cancer, the results may be skewed due to the limited number of patients. These limitations may have led to research biases that weakened the power of analysis.

## 5 Conclusions

We launched a nomogram and relating risk stratification system utilizing five clinical and treatment-related indicators to predict OS in TNAC patients. The validation of the model indicates its good predictive ability. Our nomogram provides a convenient and reliable tool for predicting the OS of TNAC patients and selecting individualized treatment.

## Author contribution

Study design: **Xiaoming Zha, Jue Wang**. Data analysis: **Yinggang Xu, Weiwei Zhang and Jinzhi He**. Data collection: **Rui Chen, Xiaoqing Shi, Xiaofeng Huang, Wenjie Shi**, **Xinyu Wan**. Drafting the manuscript:**Yinggang Xu**. Supervision of the manuscript: All authors.

## Funding

This work was supported by Chinese Society of Clinical OncologyFoundation (Y-sy2018-077, Y-JS2019-096) and 10.13039/501100001809National Natural Science Foundation of China (81302305).

## Declaration of competing interest

The authors declare no conflict of interest.

## Appendix A Supplementary data

Fig. S1 Kaplan–Meier curves showing overall survival for TNAC and TNBC patients. TNAC, triple-negative apocrine carcinoma, TNBC triple-negative breast cancer Fig. S1
