# Supplementary material for: Nomogram for predicting overall survival in patients with triple-negative apocrine breast cancer: Surveillance, epidemiology, and end results-based analysis
Source: Breast. 2022 Sep 2;66:8–14. doi: 10.1016/j.breast.2022.08.011 (PMC9465364; doi:10.1016/j.breast.2022.08.011)
Supplement: Fig. S1 — Kaplan–Meier curves showing overall survival for TNAC and TNBC patients. TNAC, triple-negative apocrine carcinoma, TNBC triple-negative breast cancer [file mmc1.docx]

Figure. S1 Kaplan–Meier curves showing overall survival for TNAC and TNBC patients.


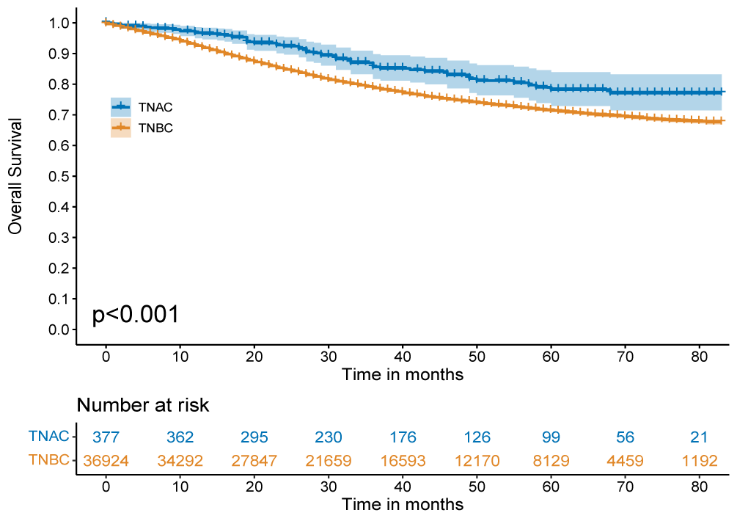


TNAC, triple-negative apocrine carcinoma, TNBC triple-negative breast cancer
